# Septic Pulmonary Emboli and Renal Abscess Caused by *Staphylococcus aureus* in an HIV-Infected Patient

**DOI:** 10.1155/2018/1460283

**Published:** 2018-01-24

**Authors:** Isaí Medina-Piñón, Alan Ledif Reyes-Mondragón, Michel Fernando Martínez-Reséndez, Adrián Camacho-Ortiz

**Affiliations:** ^1^Infectious Diseases Service, Hospital Universitario “Dr. José E. González” and Medical School, Universidad Autónoma de Nuevo León, Madero y Gonzalitos S/N, 64460 Monterrey, NL, Mexico; ^2^Department of Internal Medicine, Hospital Universitario “Dr. José E. González” and Medical School, Universidad Autónoma de Nuevo León, Madero y Gonzalitos S/N, 64460 Monterrey, NL, Mexico

## Abstract

*Staphylococcus aureus* is a common cause of bacteremia in the general population and can lead to serious metastatic infection particularly in immunocompromised persons. However, prompt diagnosis and management can result in favorable outcomes. In the following case report, the clinical course of an HIV-infected man is presented; he developed bloodstream infection (BSI) and associated complications: septic pulmonary embolism, right renal abscess, and ipsilateral renal vein thrombosis. Methicillin-resistant *Staphylococcus aureus* (MRSA) was identified as the cause of sepsis and successfully treated with surgery and antimicrobials. Intravenous vancomycin was the primary therapy, followed by oral linezolid after resolution of bacteremia.

## 1. Introduction

*Staphylococcus aureus* is a human pathogen that colonizes around 30% of the human population [1]. It is a significant cause of bacteremia worldwide, and it has been documented as the second cause of community-acquired bloodstream infections, just after *Escherichia coli* [1, 2]. Metastatic infection due to *S. aureus* can lead to septic pulmonary emboli, infective endocarditis, and septic venous thrombosis, as well as skin and soft tissue, osteoarticular, and device-related infections [2]. HIV-infected patients are more susceptible to colonization and infection caused by methicillin-resistant *Staphylococcus aureus* (MRSA), often with poor treatment response [3–5].

## 2. Case Report

A 34-year-old man was admitted to the hospital with a past medical history of herpes zoster infection one year prior to admission that was treated with oral acyclovir. He had recurrent folliculitis in both thighs, which had been treated with oral ciprofloxacin. The patient stated a history of sexual intercourse with multiple male partners without condom use.

Upon his arrival to the emergency department, he reported a 2-day history of fever, chills, and severe pain in the right-sided costovertebral angle. His vital signs showed a blood pressure of 130/80 mmHg, respiratory rate of 18 breaths/min, heart rate of 130 beats/min, and temperature of 38°C. The patient was ill-appearing, with mild dry mouth, hyperdynamic heart beats without murmurs, and tenderness in the right-sided costovertebral angle.

Initial laboratory workup revealed the following: hemoglobin 13.1 g/dL, white blood cell count 8.70 K/mm^3^, PLT 51.4 K/mm^3^; creatinine 1.4 mg/dL, albumin 2.7 g/dL, glucose 77 mg/dL, CPK 90 mg/dL, LDH 307 IU/L, AST 43 u/L, and ALT 33 u/L. Urianalysis revealed pH 5.5, 30 leucocytes/field, and proteins 215 mg/dL. Intravenous ceftriaxone was initiated 2 g daily as empiric treatment for urinary tract infection. A urine sample was collected for culture before initiation of antibiotics and was eventually reported without microbiological isolation. Positive peripheral blood cultures showed Gram-positive cocci; therefore, vancomycin was added to the antimicrobial therapy. Methicillin-resistant *Staphylococcus aureus* (MRSA) was identified by Vytek 2^®^ (bioMérieux Inc.), with a community-acquired pattern of susceptibility (susceptible antibiotics: vancomycin MIC 1 mcg/ml, clindamycin MIC <0.25 mcg/ml, trimethoprim/sulfamethoxazole MIC <10 mcg/ml, and linezolid 2 mcg/ml; resistant antibiotics: levofloxacin MIC 4 mcg/ml and oxacillin MIC >4 mcg/ml). After these results, ceftriaxone was removed.

**Figure 1:**
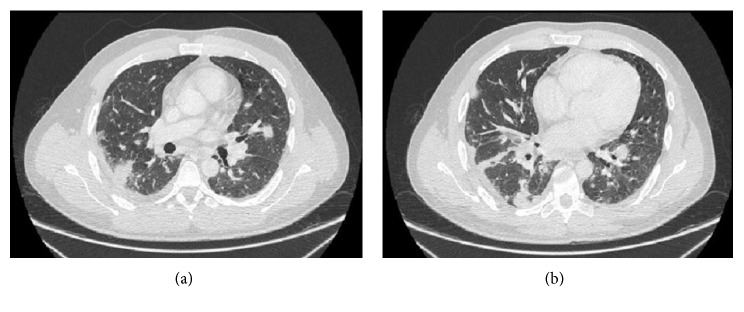
Multiple diffuse pulmonary nodules caused by *Staphylococcus aureus* septic emboli.

A contrast-enhanced thoracoabdominal computed tomography (CT) was performed, revealing multiple nodules with a bilateral and diffuse distribution, suggestive of septic emboli in the basal lung image (Figure 1). In the abdominal cavity, renal involvement was evident, showing a right-sided pyelonephritis with cortical lesions suggestive of hematogenous dissemination. Transesophageal cardiac echography demonstrated no evidence of endocarditis. Fourth-generation HIV ELISA test was positive, HIV-1 RNA viral load was 78,000 copies/ml, and CD4+ T-cell count was 80 cells/mm^3^.

**Figure 2:**
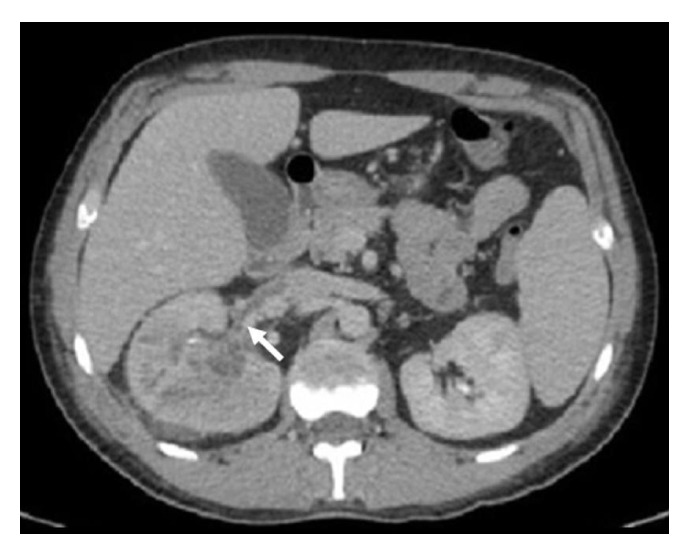
Right-sided pyelonephritis with abscess and ipsilateral renal vein thrombosis (white arrow).

The patient persisted with systemic inflammatory response syndrome (SIRS), 5 days after initiation of vancomycin at 20 mg/kg/d. New abdominal CT was performed, evidencing a right renal abscess and ipsilateral renal vein thrombosis (Figure 2). Nephrectomy was performed, and the patient improved. Culture from the renal abscess confirmed MRSA infection; subsequent blood cultures were negative. MRSA was also identified in bronchoalveolar lavage (BAL) and folliculitis biopsy cultures.

Clinical improvement was noticeable; eventually, vancomycin was switched to oral linezolid 600 mg twice daily upon discharge and continued two weeks until C-reactive protein resulted negative during outpatient follow-up. Highly active antiretroviral therapy (HAART) was initiated during his outpatient visits. After eight months of HAART, his HIV-1 RNA viral load decreased to <40 copies/mm^3^, and his absolute CD4+ lymphocytes increased to 134 cells/mm^3^. The patient has been without relapse of MRSA infection after one year of follow-up.

## 3. Discussion

The incidence of community-acquired *S. aureus* bacteremia has been estimated around 15 per 100,000 persons and a mortality rate of 3 per 100,000 persons, mainly in the Western hemisphere [3]. The incidence of *S. aureus* bacteremia among the people living with HIV is higher than that of the population not infected with HIV [1–3]. This risk is associated with the degree of cell-mediated immune deficiency, particularly in patients with CD4+ T-cell count <200 cells/mm^3^ [1, 3].

Bloodstream infections caused by *S. aureus* can be classified as “complicated” or “uncomplicated” [1]. Complicated infection is assigned when metastatic sites of infection and embolic evidence are present, as given in this case, with pulmonary and renal involvement. Other complication-related characteristics should be considered, such as community-acquired MRSA, positive follow-up blood cultures, persistent SIRS, and cardiovascular dysfunction [1, 4].

Community-acquired MRSA, compared to the hospital-acquired, can be distinguished by its decreased antimicrobial resistance pattern [5]. However, it is associated with the production of virulence factors that contribute to complications, including Panton–Valentine leukocidin (PVL), alpha-hemolysin, and the arginine catabolic mobile element (ACME) [5, 6]. These pathogen-associated molecular patterns (PAMPs) can cause persistent SIRS, exaggerated inflammation with subsequent tissue injury, and are related to genesis of septic emboli, necrotizing infection, cavitary pulmonary nodules, leucocyte lysis, thrombosis, and thrombocytopenia [1, 5–8]. Despite the setting of immunosuppression caused by HIV, a complex variety of factors have been related to the outcomes of this type of complicated bacteremia and sepsis, including impaired mechanisms of immune response, proinflammatory gene transcription inhibition, cellular dysfunction, neuroendocrine dysregulation, endothelial injury, and cardiovascular damage [8].

Septic pulmonary embolism was one of the evident complications in this case, and its physiopathology is characterized by the generation of thrombi containing microorganisms that subsequently travel through systemic veins to the right side of the heart, obstructing pulmonary vessels, and leading to septic infarction of the parenchyma [9, 10]. *Staphylococcus aureus* is the most frequent microbiological etiology, and the most common source is skin and soft-tissue infections, followed by endocarditis and thrombophlebitis [10].

Vancomycin is the recommended first-line treatment of bacteremia caused by MRSA and was the primary therapy used in this case [1, 11]. Alternative drugs for bloodstream infections caused by MRSA have been released, including telavancin, dalbavancin, oritavancin, and daptomycin; however, these options are not affordable in some developing countries [11]. Ceftaroline is the only beta-lactam antibiotic that has demonstrated activity against MRSA and is now widely used against pulmonary and soft-tissue infections. Although it has not been FDA-approved for bloodstream infections, off-label prescription as salvage therapy has shown to be a promising alternative in monotherapy or combined therapy [12, 13].

In conclusion, bloodstream infection associated with MRSA is a serious disease that can be a diagnostic and therapeutic challenge. HIV-infected patients are more susceptible than the general population, and the morbidity and mortality rates can be reduced with multidisciplinary management, following the treatment principles including prompt identification and removal of source, exclusion of possible metastases, and prescription of the antibiotic treatment.
